# Radiosensitization of Allogenic Subcutaneous C6 Glioma Model with Focused Ultrasound-Induced Mild Hyperthermia

**DOI:** 10.3390/life14030359

**Published:** 2024-03-09

**Authors:** Zhiyuan Xu, David Schlesinger, Robert Andrew Drainville, David Moore, Patcharin Pramoonjago, Jason Sheehan, Frederic Padilla

**Affiliations:** 1 Department of Neurological Surgery, University of Virginia Health System, Charlottesville, VA 22903, USA; zx5b@hscmail.mcc.virginia.edu (Z.X.); djs9c@hscmail.mcc.virginia.edu (D.S.); jps2f@uvahealth.org (J.S.); 2 LabTAU, INSERM, Centre Léon Bérard, Université Lyon 1, F-69003 Lyon, France; andrew.drainville@inserm.fr; 3 Focused Ultrasound Foundation, Charlottesville, VA 22903, USA; dmoore@fusfoudantion.org; 4 Department of Pathology, University of Virginia Health System, Charlottesville, VA 22903, USA; pp6f@hscmail.mcc.virginia.edu; 5 Department of Radiology, University of Virginia Health System, Charlottesville, VA 22903, USA

**Keywords:** hyperthermia, radiotherapy, radiosensitization, glioma, focused ultrasound

## Abstract

The radiosensitization potential of focused ultrasound (FUS)-induced mild hyperthermia was assessed in an allogenic subcutaneous C6 glioma tumor model in rats. Mild hyperthermia at 42 °C was induced in tumors using a single-element 350 kHz FUS transducer. Radiation was delivered with a small animal radiation research platform using a single-beam irradiation technique. The combined treatment involved 20 min of FUS hyperthermia immediately before radiation. Tumor growth changes were observed one week post-treatment. A radiation dose of 2 Gy alone showed limited tumor control (30% reduction). However, when combined with FUS hyperthermia, there was a significant reduction in tumor growth compared to other treatments (tumor volumes: control—1174 ± 554 mm^3^, FUS-HT—1483 ± 702 mm^3^, 2 Gy—609 ± 300 mm^3^, FUS-HT + 2 Gy—259 ± 186 mm^3^; ANOVA *p* < 0.00001). Immunohistological analysis suggested increased DNA damage as a short-term mechanism for tumor control in the combined treatment. In conclusion, FUS-induced mild hyperthermia can enhance the effectiveness of radiation in a glioma tumor model, potentially improving the outcome of standard radiation treatments for better tumor control.

## 1. Introduction

Current therapeutic options for intracranial tumors often center on surgery or stereotactic radiosurgery (SRS), with the latter in particular demonstrating good control rates with low treatment toxicity [1,2,3]. The standard treatment for glioblastoma multiforme (GBM) is surgical resection followed by chemoradiotherapy, with radiotherapy usually distributed over 6 weeks in 2 gray (Gy) fractions for a total dose of 40–60 Gy, or more focally with SRS in fewer fractions and at higher doses per fraction (15 + Gy) [1]. However, many brain tumors are less amenable to a favorable radiation response, including diffuse tumors such as GBM [3] and tumors with areas of central necrosis and hypoxia [4]. Because of the infiltrative nature of GBM, regional recurrence of the initial tumor remains a potent cause of relapse [5].

Hyperthermia (HT) has been reported to sensitize various tumor types to radiation [6,7]. Clinical evidence showed the safety and feasibility of combining various hyperthermia modalities with radiotherapy (RT) for high-grade glioma (HGG) patients [6]. In the single phase III study randomized controlled trial evaluating combined HT + RT compared to RT alone in HGG published so far [7], the addition of HT significantly prolonged survival in patients treated after surgery with RT and interstitial brachytherapy. The clinical application of HT for brain tumors, however, has been hampered to date by the lack of safe and efficient technology to produce localized, low-level hyperthermia.

This situation presents an opportunity for focused ultrasound FUS-induced hyperthermia (FUS-HT) to be applied as an adjuvant treatment, enhancing the primary response to RT. Several clinical studies have shown that FUS-HT, when used in conjunction with RT, is well tolerated and exhibits minimal cytotoxicity [8]. It has proven to be an effective supplement, enhancing tumor control and response rates in a variety of malignancies [8,9,10,11,12,13]. Furthermore, the feasibility of FUS-induced hyperthermia has been demonstrated in an intracranial preclinical tumor model [14].

FUS-induced HT was clinically tested as an adjunct to RT for brain tumor treatment as early as 1991 in a phase I study [15]. Its adoption was initially slow due to challenges such as the need for craniectomy and invasive temperature probes [15]. However, the recent development of magnetic resonance-guided focused ultrasound (MRgFUS), which is currently used clinically for treating essential tremor [16], has renewed interest in FUS-HT + RT combination for treating brain tumors as it enables brain treatment without craniotomy and with real-time MR thermometry and temperature monitoring [6].

We hypothesize that RT + FUS-induced hyperthermia (FUS-HT) will permit the localized radiosensitization of a radiosurgical target. In the study presented here, we applied FUS-HT with direct temperature measurement using a thermocouple and investigated differences in outcomes with radiotherapy alone, FUS-induced hyperthermia alone, combined radiotherapy, and FUS-induced hyperthermia (FUS-HT) in a preclinical model of glioma implanted subcutaneously. We hypothesized that combined RT + FUS-HT would improve tumor growth control. We also qualitatively investigated the mechanisms of the combination by the induction of DNA damage.

## 2. Materials and Methods

### 2.1. Experimental Design

For the radiation sensitivity assessment aimed at identifying a suboptimal radiation dose, animals were randomized into three groups (*n* = 3 per group): untreated, RT 2Gy, and RT 5Gy. For the radiosensitization experiments, designed to assess the tumor growth control provided by different treatment modalities, the animals were randomized into four groups: untreated (*n* = 8), RT 2Gy (*n* = 8), FUS-HT (*n* = 10), and FUS-HT + RT 2Gy (*n* = 10).

### 2.2. Allogenic Subcutaneous C6 Glioma Tumor Model

Animal experiments were approved by the Institutional Animal Care and Use Committee (ACUC) at the University of Virginia. A subcutaneous C6 rat glioma model [17] was used. Eight-week-old female Wistar rats received subcutaneous inoculation of 3 × 10^6^ C6 cells (ATCC) cells. Rats were randomized into treatment groups 4–5 days post-implantation, when tumors were palpable and 5 to 6 mm in diameter. Tumor size was recorded bi-daily over the week following treatment utilizing a digital caliper. Tumor volume (V) was estimated from two orthogonal measurements, length (the longest dimension) and width (the dimension perpendicular to length), as V = ½ (Length × Width^2^) [18]. To minimize variability, a single operator conducted all measurements for consistency. Additional clinical signs, including changes in animal weight and behavior, were also considered.

### 2.3. Small Animal Radiation Treatment

Animals were treated with 2 Gy or 5 Gy in a single fraction delivered to the center of the tumor using a Small Animal Radiation Research Platform or SARRP (XStrahl, Camberely, UK). The tumor (visible as a perturbation underneath the skin) was aligned with the targeting lasers of the SARRP system. The skin surface of the animal immediately superficial to the tumor was placed at the level of the horizontal targeting laser. Image guidance was not used (or required) for targeting. The animal body, except the tumor, was shielded with lead apron. Irradiation times (0.6 min for 2 Gy or 1.5 min for 5 Gy) were calculated according to the desired dose for the tumor, the base output dose rate of the SARRP (~4.1 Gy/min), the estimated depth of the tumor (approximately 3 mm), and the output factor of the 5 mm collimator (~0.79). For combination therapy, rats underwent FUS hyperthermia for 20 min, followed by radiation treatment within 20 min.

### 2.4. FUS Hyperthermia Experimental Setup

FUS hyperthermia was induced with a focused, single-element 350 kHz transducer (diameter of 44 mm, focal length of 31.5 mm, focus dimensions at −3 dB are 3.1 mm in width, 15.4 mm in length, model H224, Sonic Concept, Bothell, WA, USA), connected to a power amplifier (E&I 1020 L, Rochester, NY, USA) and function generator. The pressure field of the focused 350 kHz transducer was calibrated using a needle hydrophone (HNA-0400, Onda Corp., Sunnyvale, CA, USA).

The animals were positioned on a customized 3D-printed PLA holder so that the center of the tumor was at the focal point of the transducer. Coupling along the acoustical axis was provided by using ultrasound coupling gel and a gel pad (Aquaflex, Parker Laboratories, Fairfield, NJ, USA). Temperature was monitored with a 0.3 mm needle thermocouple (HYP1-30-1/2-T-G-60-SMP-M, Omega, Biel/Bienne, Switzerland) inserted in the center of the tumor (positioning was verified by portal ultrasound device (Chison ECO1, Chison Medical Technologies Co., Ltd., Chison, Jiangsu, China) and connected to a Proportional–Integral–Derivative (PID) controller to regulate the temperature to 42 °C. The PID controller was connected to the external trigger of a function generator to provide an ON signal when the temperature was below the target temperature and an OFF signal when the temperature was above the target temperature.

The Ispta intensity, spatial peak temporal average, was estimated as P_max_^2^/2rc, with Pmax as the peak pressure, c as the speed of sound in water, and r as the density of water. Because the system is operated with CW, Ispta, and Isppa, spatial peak pulse average intensities are equivalent.

### 2.5. Simulations of Treatment Parameters

**Figure 1:**
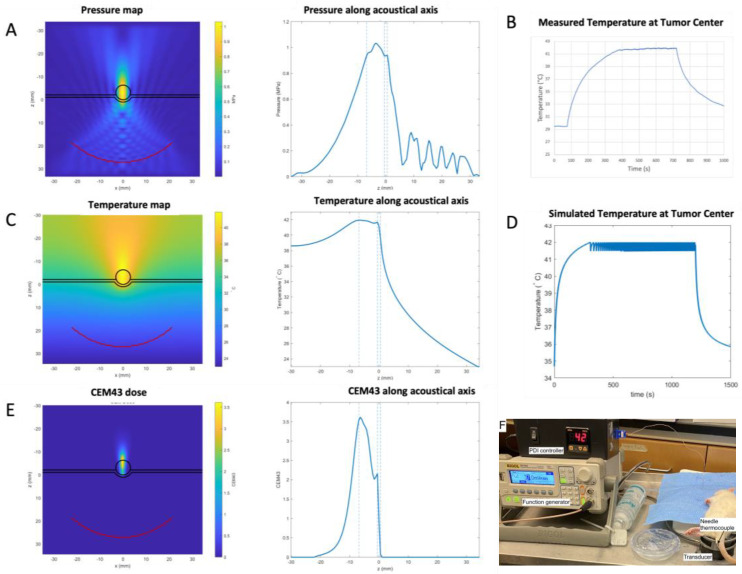
Numerical simulation of FUS propagation and the resulting temperature increase in tumors. (**A**) Left: simulation model composed of water, a skin layer (0.8 mm thick), a tumor (5 mm diameter), and soft tissues, with a superposition of the k-Wave simulation of acoustic pressure field from the focused 350 kHz transducer emitting a 1 MPa peak pressure in CW at focus, used as a heat source in BHTE simulations. Right: simulated pressure along the acoustical axis of the transducer, with dashed lines delimiting skin and tumor boundaries. (**B**) Temperature regulation in a tumor using the PID controller. For this proof-of-concept experiment, temperature was maintained at 42 °C for 5 min. (**C**) Left: simulated temperature distribution in the tumor. Right: simulated temperature along the acoustical axis of the transducer. (**D**) Simulated temperature over time at the center of the tumor, with an ON/OFF command of the transducer once the target temperature of 42 °C is reached. For these simulations, the values listed in Table 1 were used. (**E**). Left: estimated CEM43 distribution in the tumor. Right: estimated CEM43 along the acoustical axis of the transducer. (**F**) Image of the experimental set-up, including the transducer, the PID controller, and the thermocouple.

The acoustic pressure required to induce hyperthermia was first evaluated by 3D numerical simulations using the k-Wave MATLAB R2021a toolbox [19] to characterize the acoustic field produced within the tumor and surrounding tissue, coupled with a numerical bioheat equation (BHTE) solver, custom-written with MATLAB, to predict temperature elevation (Figure 1). The material configuration used in simulations was constructed to approximate a 6 mm diameter tumor situated just beneath a thin layer of skin of 0.8 mm thickness and coupled with water to mimic the coupling gel used in the experiments, as illustrated in Figure 1. The material properties used in simulations are shown in Table 1. The transducer, with a diameter of 44 mm and a focal length of 31.5 mm, was positioned such that the geometric focus was located at the tumor center. The transducer emitted in CW mode, as in the experiments. The numerical BHTE simulations were then performed using the same material configuration and grid used for acoustic simulations. The heat source term was obtained based on the acoustic amplitude obtained from pressure field simulations. The temperature values were initialized with values of 37 °C for biological tissue and 23 °C for water. The temperature was fixed at the boundaries perpendicular to the acoustical propagation axis z in water, and all other boundary conditions were considered perfectly isolating. The temperature was simulated for 10 min before the heat source term was applied, to allow the temperature to approach an equilibrium in the different media. The temperature at the tumor center was monitored, with the heat source being disabled when the temperature exceeded 42 °C and again being enabled when the temperature fell below 41.5 °C to mimic the behavior of the PID controller used to switch ON/OFF the output of the function generator during experimental treatment. The Cumulative Equivalent Minutes at 43 °C (CEM43) dose was calculated using the simulated temperature maps [20].

### 2.6. Immunohistochemistry

Animals were euthanized 24 h after treatment, and tumors were harvested and stored in 10% NBF (Sigma, St. Louis, MO, USA) overnight and then processed for immunohistochemistry using gamma H2AX (Novus Biologicals, Centennial, CO, USA, NB100-417) at 1:400 dilution. Images were obtained using a NanoZoomer S360 platform from Hanamatsu (Hamamatsu City, Japan) at ×20. Gamma-H2AX, a biomarker for DNA double-strand breaks, forms detectably within minutes of ionizing radiation exposure and typically diminishes within 24 h due to cellular repair mechanisms. It is imperative to perform assessments at this relatively early time point, as cells sustaining significant damage are likely to be dead by one week, rendering gamma-H2AX an irrelevant marker for DNA damage at later time points. Given this, a 24 h post-treatment mark was selected to provide a pertinent time point to assess the presence of irreparable damages.

### 2.7. Statistical Analyses

Tumor volumes are expressed as the mean ± standard deviation. Data were analyzed using R (version 3.6.1). Comparisons of treatment groups were performed by analysis of variance (ANOVA), followed by Welch’s two-sample *t*-test with a Bonferroni correction, with *p* values of 0.05 indicating statistical significance.

## 3. Results

### 3.1. Treatment Parameters as Determined by Numerical Simulations

Parametric 3D numerical simulations using the k-Wave MATLAB toolbox determined that 1 MPa peak acoustic pressure could elevate tumor temperature to 42 °C within a few minutes (Figure 1) and maintain it during the treatment time of 20 min by positioning a single focus of our focused transducer at the center of the lesion. Hyperthermia and temperature regulation with the PID controller were validated in animals bearing tumors (Figure 1), with a proof-of-concept experiment where the temperature was maintained at 42 °C for 5 min, as predicted by the k-Wave simulations in tissues.

BHTE simulations revealed a rather uniform temperature elevation within the tumor. Based on the maximum simulated CEM43 value of 3.5, below a tissue damage threshold in mice [25], no irreversible damage to healthy soft tissues was anticipated. Given a peak pressure of 1 MPa, the estimated Ispta was estimated to be 34 W/cm^2^. No animals showed signs of adverse effects after hyperthermia treatments and an examination of the animal’s skin showed no signs of skin damage, including no trace of skin burns.

### 3.2. Allogenic Subcutaneous C6 Tumor Models Sensitivity to Radiation

**Figure 2:**
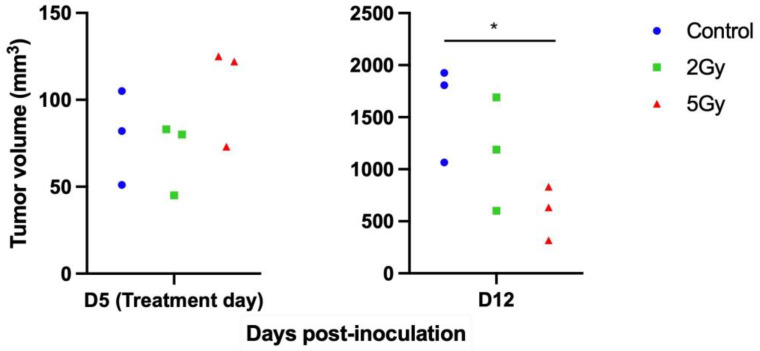
Allogenic subcutaneous C6 tumor models response to radiotherapy. Tumors were treated on day 5 post-implantation with either sham treatment (Control) or a single dose of 2 Gy or 5 Gy. At one week post-treatment, the mean tumor volumes were 1657 ± 397 mm^3^, 1160 ± 545 mm^3,^ and 595 ± 259 mm^3^ for the control (untreated, in blue), 2 Gy (in green) and 5 Gy (in red) groups, respectively. *n* = 3 per group. * indicate a *p* < 0.05.

A dose-escalation study showed that within a week, 5 Gy and 2 Gy RT reduced tumor volume by 65% and 30% compared to untreated animals, respectively (Figure 2). Only the reduction between the control and 5 Gy groups was significant (*p* < 0.05). The 2 Gy dose was found to be suboptimal to control tumor growth. Treating tumors with a high radiation dose of 5 Gy resulted in significant regression, preventing a discernible opportunity to evaluate the potential synergistic effect when combined with FUS-induced hyperthermia. Consequently, a 2 Gy dose was selected for assessing the radiosensitization provided by FUS-HT in the combined treatment and to mirror the clinical scenario wherein the tumor exhibits partial to no response to radiation therapy.

### 3.3. Efficacy of FUS-Hyperthermia and RT

**Figure 3:**
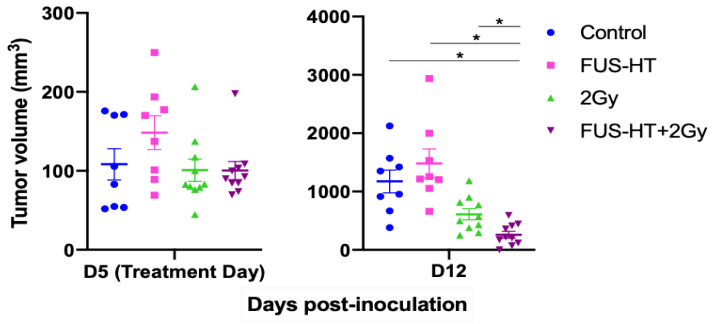
Subcutaneous C6 tumor models response to RT (2 Gy), FUS hyperthermia (FUS-HT) or a combination of FUS-RT and RT. Tumors were treated on day 5 post-implantation, and they were monitored over the course of one week post-treatment. N = 8 to 10 per group. There was no significant difference between tumor volumes on the treatment day (mean tumor volumes on treatment day 108 ± 56 mm^3^ for control, 101 ± 45 mm^3^ for 2 Gy, 134 ± 48 mm^3^ for FUS-HT, 100 ± 36 for FUS-HT + 2 Gy, ANOVA *p* value = 0.45). * denotes *p* < 0.05.

There was no significant difference in tumor volumes between groups on the treatment day (ANOVA *p* value 0.45, Figure 3, left). One week post-treatment (Figure 3, right), significant differences were observed among the treatment groups (mean tumor volumes one week post-treatment 1174 ± 554 mm^3^ for control, 1483 ± 702 mm^3^ for FUS-HT, 609 ± 300 mm^3^ for 2 Gy, 259 ± 186 mm^3^ for FUS-HT + 2 Gy, ANOVA *p* value < 10^−5^). FUS-HT alone or RT 2 Gy alone did not confer statistically significant tumor growth control compared to untreated tumors (Welch *t*-test *p* > 0.1), despite a trend in tumor volume reduction following RT 2 Gy alone. However, combined FUS-HT and 2 Gy RT treatment resulted in significant tumor growth control improvement compared to other modalities (Welch *t*-test *p* = 0.012 compared to untreated, *p* = 0.04 compared to 2 Gy, *p* = 0.01 compared to FUS-HT).

After the combined treatment, where a lead shield was used to protect the soft tissues around the target area during the radiation process, potential damage to the soft tissues was assessed by macroscopic examination when the tumor was removed. No signs of damage were found, and the skin also remained intact post-treatment.

### 3.4. Effect of Treatments on DNA Damage Repair Mechanism

**Figure 4:**
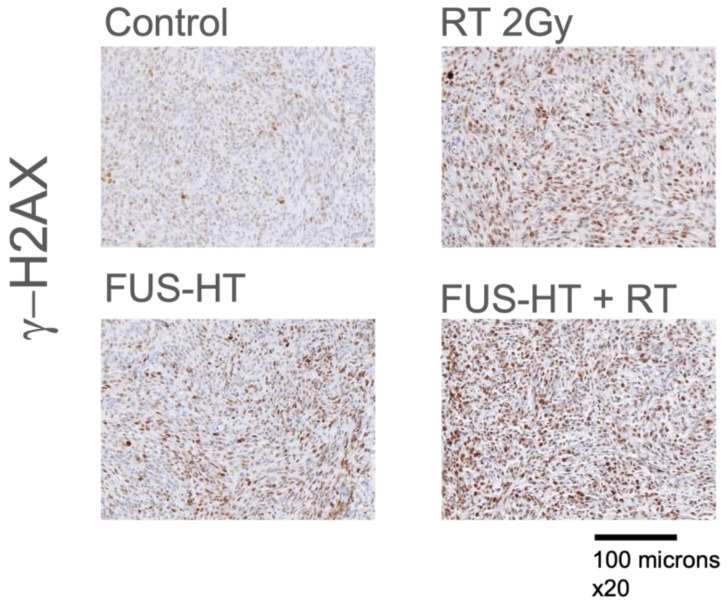
Representative images of immunohistochemistry of tumors 24 h post-treatment. DNA double-strand break damage was detected by H2AX (γ-H2AX) immunohistochemistry.

The DNA damage potential of combined FUS-HT + RT treatment was assessed via g-H2AX staining 24 h post-treatment. Figure 4 shows representative images. Qualitatively, the combined treatment had a higher proportion of cells with DNA damage compared to individual modalities or untreated tumors.

## 4. Discussion

The recent development of magnetic resonance–guided FUS (MRgFUS) can now allow for a noninvasive approach to perform HT or thermal lesioning to brain tumors with volumetric thermometry.

FUS hyperthermia and subablative radiation together can effectively limit tumor growth in a subcutaneous glioma model. Using a single focal zone, hyperthermia at 42 °C can be induced and maintained without damage to untargeted tissues or skin. Because temperature measurement by thermocouple probes during ultrasound-induced hyperthermia can be prone to errors [26,27], potential errors in temperature measurement were assessed using a “wait and see” approach [28]. No significant artifacts were observed at the very moderate intensity used below 35 W/cm^2^ Isppa, typical for FUS-induced hyperthermia [29]. Although the transducer focal area did not encompass the entire tumor, as illustrated by the simulations in Figure 1, the BHTE simulations revealed a rather uniform temperature distribution within the tumor due to thermal diffusion.

FUS hyperthermia alone does not control tumor growth; however, when combined with a radiation sub-optimal to control tumor growth, it significantly improves tumor growth control. While the optimal radiation–heat sequence in clinical hyperthermia remains to be clarified for this indication, previous studies suggest that heat and radiation are to be applied within a short period of time to optimize the potentiation effect, with a longer period of time available for the heat–radiation sequence than for the radiation–heat sequence [30]. The maximum 20 min interval between hyperthermia and radiation was necessary because of logistical constraints, specifically the time required to transport the animals from the FUS treatment room to the facility housing the small animal radiator. Although the temperature inside the tumors might decrease during this time interval, it was reported that supra-additive effects of heat and radiation may occur for a period of time up to a couple of hours [30], much longer than the contained 20 min interval used in this study.

Tumor volume measurements were recorded bi-daily over the week following treatment. At greater follow-up times, subcutaneous C6 tumors spontaneously regress in size [31], most likely because of the model’s allogenic characteristics in Wistar rats [32]. A standard methodology was employed for monitoring tumor growth, utilizing a digital caliper to take two orthogonal measurements, length, and width, of the tumors. The caliper’s precision was 0.01 mm. The caliper method, widely accepted in preclinical oncology studies, offers a reliable means to monitor tumor growth [18]. However, caliper measurements assume an ellipsoidal tumor shape for volume estimation from 2D measurements, which may not be as accurate as 3D imaging modalities, such as ultrasound [33] or MRI [34]. A single operator conducted all measurements for consistency and to minimize variability. Another potential source of minor measurement variation could be tumor pseudo-regression, which might result from treatment-induced swelling or inflammation. Clinical signs were closely monitored to exclude this possibility.

One of the reported sensitization mechanisms of hyperthermia is damaged DNA-repair machinery [35]. Short-term analysis of treated tumors showed qualitatively increased DNA damage. Other mechanisms might be contributory, particularly at different time intervals, resulting in damage to DNA repair mechanisms, overexpression of heat shock proteins (HSP), or in increased blood flow and oxygenation of the tumor, as these are reported hyperthermia sensitization mechanisms [35].

While our research focuses on GBM, we utilized a subcutaneous inoculation of the C6 tumor cell line, a model that does not encompass a blood–brain barrier (BBB). This approach was strategically chosen to first evaluate the therapeutic potential in a more accessible tumor environment, laying the groundwork for future investigations. The widely used C6 glioma model has limitations, and further studies should assess the treatment’s effectiveness in intracranial tumor models with a functioning BBB, optimizing treatment doses and timing. For clinical application, the exploration of more sophisticated exposure schemes and feedback control systems, such as MRgFUS hyperthermia, is required [36].

Intracranial FUS-HT has been reported in preclinical models with preclinical FUS systems [14,36], and the feasibility of hyperthermia with MRg-FUS clinical systems has also been reported in tissue phantoms [29,37]. There are still constraints to overcome, primarily due to limitations in the treatment envelope and the extended duration required for effective treatment, but intracranial FUS-HT is technically feasible, although not yet deployed in clinical systems.

Only two doses of radiation, 2 and 5 Gy, were tested in this study. This decision on dosage was primarily based on the existing literature [38]. Given the complexities inherent in these preclinical experiments, a comprehensive examination of responses to various RT doses was not feasible. The primary goal was to identify both an ablative and a sub-ablative dose. Based on the experimental results, 5 Gy and 2 Gy were validated to serve these roles, respectively.

The acoustic parameters, 1 MPa peak pressure corresponding to an Ispta of 34 W/cm^2^, were specifically chosen to maintain a temperature of 42 °C. One constraint in the experimental setup used in the study comes from the PID controller, which necessitates the use of constant acoustic pressure as the regulation mechanism operates by turning the transducer’s emission ON or OFF. More sophisticated schemes could be implemented, such as adjusting the level of acoustic pressure. Nevertheless, this straightforward approach achieved and sustained mild hyperthermia, enabling the examination of its combination with RT.

In conclusion, our exploratory study demonstrates that FUS-induced mild hyperthermia, verified via direct temperature measurements in tissue, has the potential to enhance the radiosensitivity of a rat glioma model to radiation, achieving effective tumor growth control with a subablative radiation dose. This promising finding underscores the value of combining radiotherapy with ultrasound-induced hyperthermia as a therapeutic strategy for brain tumors. Moving forward, it will be essential to validate these results in an intracranial tumor model and to investigate the efficacy of different treatment sequences to optimize therapeutic outcomes.

## Figures and Tables

**Table 1:** Parameters used for the FUS numerical simulations. Values for the different tissue properties were taken from [21,22,23]. The thickness of the rat skin was set to 0.8 mm following [24].

Parameter	Units	Water	Skin	Tumor	Muscle
Density	kg/m^3^	1000	1060	1060	1060
Speed of sound	m/s	1482	1624	1560	1588
Attenuation	Np/(m·MHz)	0	21.2	9.32	7.1
Thermal Conductivity	W/(m·°C)	0.627	0.5	0.5	0.52
Specific Heat	J/(kg·°C)	4118	3700	3700	3700
Perfusion	kg/(m^3^·s)	0	2.0	20	0.7

## Data Availability

The data supporting the findings of this study can be accessed upon reasonable request.
